# Immersive virtual reality for teaching hemoglobin structure in preclinical medical biochemistry education: a mixed-methods study of student self-reported perceptions

**DOI:** 10.1186/s12909-026-08736-4

**Published:** 2026-02-03

**Authors:** Iman Dajani, Maria Christina Esteban, Ali Chaari

**Affiliations:** Weill Cornell Medicine Qatar, Qatar Foundation, P.O. Box 24144, Education City, Doha, Qatar

**Keywords:** Virtual reality, Biochemistry education, Hemoglobin, Molecular visualization, Medical curriculum, Nanome, Student engagement, Spatial learning

## Abstract

**Background:**

Understanding protein structure is foundational to preclinical medical education and underpins later learning in hematology, physiology, and pharmacology. However, many biochemical concepts, particularly those involving three-dimensional molecular architecture, are difficult for students to conceptualize using traditional two-dimensional instructional tools. Immersive virtual reality (VR) enables interactive, stereoscopic visualization of biomolecules and may support spatial understanding of complex protein structures. This study aimed to evaluate undergraduate preclinical medical students’ self-reported perceptions of learning, spatial understanding, and engagement following a VR-based instructional session focused on hemoglobin structure.

**Methods & data analysis:**

In this mixed-methods study, a retrospective within-session pre/post evaluation was embedded within a preclinical medical biochemistry course at Weill Cornell Medicine-Qatar. Students used the Nanome platform on standalone VR headsets to explore hemoglobin’s quaternary structure, heme coordination, and conformational transitions. Quantitative outcomes were assessed using three 10-point Likert-scale items administered before and after the session. Qualitative data were collected through open-ended survey questions and analyzed thematically.

**Results:**

Fifty-four students participated. Following the VR session, a larger proportion of students selected the highest rating categories for perceived usefulness of VR (63%), spatial understanding of hemoglobin (66.7%), and overall structural understanding (70.5%). Overall responses reflected consistently positive self-reported perceptions of learning and visualization. Mild discomfort related to headset use was reported by a minority of participants (35 none, 13 transient, 6 persistent). Qualitative feedback highlighted clearer subunit differentiation, improved visualization of the heme environment, and collaborative discussion.

**Conclusions:**

VR-based molecular visualization was associated with consistently positive student-reported perceptions of spatial understanding and engagement in preclinical biochemistry education. These findings suggest that immersive VR may serve as a feasible adjunct to conventional instructional methods for teaching spatially complex molecular concepts.

**Supplementary Information:**

The online version contains supplementary material available at 10.1186/s12909-026-08736-4.

## Highlights

• Virtual reality provides an immersive tool to teach complex biochemical structures.

• Integration of VR into a medical biochemistry curriculum was feasible and well received.

• Immersive molecular modeling promoted deeper spatial understanding and engagement.

• Student feedback highlighted enhanced visualization, interactivity, and collaboration.

• VR complements traditional methods and supports wider curricular integration.

**Supplementary Information:**

The online version contains supplementary material available at 10.1186/s12909-026-08736-4.

## Background

 Biochemistry is a foundational component of medical education, underpinning subsequent learning in physiology, pathology, pharmacology, and clinical medicine. Despite its central role, biochemistry is consistently reported as one of the most conceptually challenging domains for undergraduate and preclinical medical students, particularly due to its reliance on abstract molecular reasoning and integration across multiple scales of biological organization. Recent systematic reviews of biochemistry education in higher education highlight persistent difficulties in student comprehension and conceptual integration, even when diverse instructional strategies are employed, underscoring the need for pedagogical approaches that better support complex cognitive demands [1].

Across higher education and preclinical medical curricula, educators employ a range of instructional modalities to support learning of complex scientific content, including didactic lectures, problem-based learning, laboratory instruction, and digitally supported visualization tools. Large-scale syntheses of undergraduate STEM education demonstrate that these modalities are often combined to address conceptual difficulty and promote deeper understanding, although their effectiveness depends strongly on how they support cognitive engagement rather than on modality alone [2]. Within biochemistry education, however, contemporary reviews indicate that many commonly used approaches continue to rely heavily on two-dimensional (2D) representations, which may inadequately support spatial reasoning and mental reconstruction of three-dimensional (3D) molecular structures [3, 4].

Virtual reality (VR) has emerged as a promising instructional modality for addressing these spatial and cognitive challenges. The adoption of virtual reality technologies in education has increased globally, particularly within medical, biological, and laboratory-based training contexts. Recent systematic and scoping reviews document expanding implementation of immersive VR tools to support visualization of complex spatial concepts and learner engagement across higher education settings [3, 5].

However, despite this growth, much of the existing literature continues to emphasize learner perceptions, feasibility, and satisfaction rather than controlled evaluations of learning outcomes, highlighting an ongoing gap in rigorous educational evidence [1, 6]. Additionally, these evidence syntheses emphasize substantial heterogeneity in study designs, outcome measures, and implementation strategies, highlighting that VR’s educational value depends less on technological novelty and more on thoughtful pedagogical integration and context-specific evaluation [3, 7].

Within biochemistry education specifically, systematic reviews identify molecular structure-function relationships as a recurring barrier to student understanding, particularly when instruction relies on static or symbolic representations of inherently dynamic systems [1]. Hemoglobin exemplifies this challenge: its quaternary structure, cooperative oxygen-binding behavior, and conformational transitions require learners to integrate spatial, chemical, and functional information simultaneously. While a variety of digital and physical visualization tools have been explored for teaching protein structure, recent evidence syntheses continue to call for instructional approaches that support embodied spatial interaction and collaborative exploration of molecular systems in preclinical settings [4, 5].

In response to these identified gaps, the present study evaluates the use of an immersive VR molecular visualization platform within a preclinical medical biochemistry course. Rather than assessing objective learning outcomes, this mixed-methods study focuses on undergraduate medical students’ self-reported perceptions of spatial understanding, conceptual clarity, and engagement following a structured VR-based instructional session centered on hemoglobin structure. By situating this work within contemporary evidence syntheses and responding to calls for context-specific educational evaluation, the study aims to contribute to the growing literature on immersive learning tools in medical education while maintaining appropriate methodological scope and interpretive caution.

## Methods

### Study design & participants

This pilot study was conducted at Weill Cornell Medicine-Qatar (WCM-Q) and involved 54 undergraduate medical students enrolled in the core biochemistry course during the Spring 2024 semester. The study aimed to evaluate whether using VR in combination with the Nanome software platform would improve students’ spatial and conceptual understanding of hemoglobin structure. Participation was integrated as part of a scheduled teaching session and was reviewed and deemed exempt by the Institutional Review Board of Weill Cornell Medicine-Qatar (IRB# 2326289-2).

Participation in the VR activity occurred during a scheduled instructional session within the core biochemistry course. All students enrolled in the course during the Spring 2024 semester were eligible to participate in the VR session as part of standard teaching activities. Participation in the research component, including completion of the pre- and post-activity surveys, was voluntary. Students were informed of the study purpose in class and invited to participate at the start of the session; no additional recruitment methods (e.g., email invitations or external advertisements) were used.

The study cohort therefore represents a convenience (non-probability) sample of undergraduate medical students enrolled in a single preclinical biochemistry course. No remuneration, academic credit, grade incentives, or other rewards were provided for participation in either the VR activity or the associated surveys.

Inclusion criteria consisted of enrollment in the core biochemistry course and attendance at the scheduled VR teaching session. No formal exclusion criteria were applied. All students who attended the session were able to participate in the VR activity, and all participants completed the pre- and post-activity Likert-scale survey items.

Students who routinely used vision correction (glasses or contact lenses) were permitted to wear their corrective lenses during the VR session. Vision correction status was not recorded as a study variable, and no participants reported being unable to engage with the VR activity due to visual limitations.

### Activity procedure

The VR session lasted approximately 35 min per group of students and was divided into three distinct segments. Each component was structured to enhance student understanding of hemoglobin structure and function using immersive molecular visualization, peer engagement, and conceptual reinforcement through discussion.

#### Orientation & interface familiarization (10 min)

Each student used a dedicated standalone virtual reality headset (Meta Quest 2; Meta Platforms, Inc., Menlo Park, CA, USA) running *Nanome* (Nanome Inc., San Diego, CA, USA; version current at the time of the study), a molecular visualization platform that enables interactive manipulation and collaborative exploration of three-dimensional molecular structures in immersive virtual reality. The VR application was run directly on the headset using its native processing hardware; no external desktop or laptop computer was required to host or stream the VR environment during the session. Prior to each session, the quaternary structures of hemoglobin were preloaded into Nanome using data obtained from the Protein Data Bank (PDB). The structures selected were deoxyhemoglobin (PDB ID: 1A3N) and oxyhemoglobin (PDB ID: 6BB5), allowing students to examine molecular changes associated with oxygen binding.

**Fig. 1:**
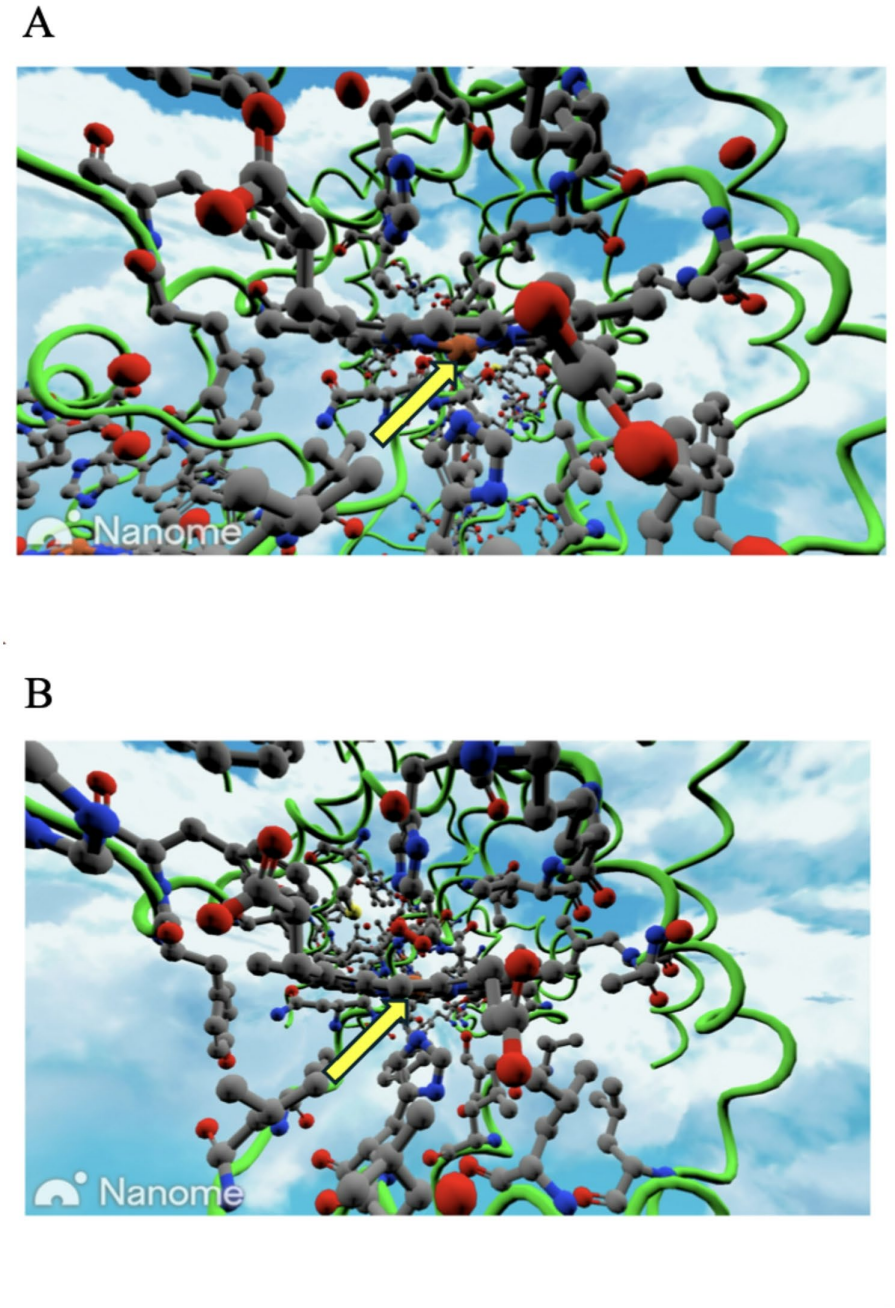
Nanome-rendered visualization of hemoglobin states used in the VR tutorial. Panel **A** shows deoxyhemoglobin (T state, PDB ID: 1A3N), where the Fe²⁺ ion (brown) lies out of the porphyrin plane, coordinated by the proximal histidine (His F8). Panel** B** shows oxyhemoglobin (R state, PDB ID: 6BB5), where oxygen binding pulls Fe²⁺ into the plane, shifting the proximal histidine and initiating the cooperative T→R transition. Students compared these states to identify key residues, visualize the role of proximal and distal histidines, and link heme-level changes to quaternary structural rearrangements

Before entering the VR environment, students received a brief 10-minute orientation session. This included a focused review lecture on the structural organization of hemoglobin, cooperative oxygen binding, and clinical significance. Students were introduced to Nanome’s core interactive features, such as molecular manipulation (rotation, zoom, translation), toggling visualization styles (wireframe, surface, ribbon), and selecting specific atoms or residues. The tutorial emphasized the identification of key structural components, including the heme prosthetic group, the coordinated Fe²⁺ ion, and the proximal and distal histidines, all of which were visually highlighted and discussed using the dual-state molecular visualization of deoxyhemoglobin and oxyhemoglobin (Fig. 1A). This onboarding ensured that students were confident in both spatial navigation and residue-level molecular interpretation prior to collaborative engagement.

#### Hemoglobin structural analysis (15 min)

The explanatory content related to hemoglobin structure, cooperative oxygen binding, and allosteric regulation was delivered prior to the immersive VR activity as part of the orientation and interface familiarization session described in Sect. 2.2.1. The second session occurred in small groups, typically comprising two students and one instructor in a shared virtual Nanome workspace. Within this immersive setting, students collaboratively explored hemoglobin’s tetrameric structure, examining the spatial orientation and quaternary arrangement of the α and β subunits. Guided by the instructor, students analyzed structural differences between the T (tense, deoxygenated) state and the R (relaxed, oxygenated) state, paying particular attention to conformational shifts that occur upon oxygen binding (Fig. 1B). Students located and compared the proximal and distal histidines, observed the positional change of the Fe²⁺ ion relative to the heme plane, and discussed how these local shifts influence global structural rearrangements at the α₁-β₂ and α₂-β₁ interfaces, leading to narrowing of the central cavity and enhanced oxygen affinity.

**Fig. 2:**
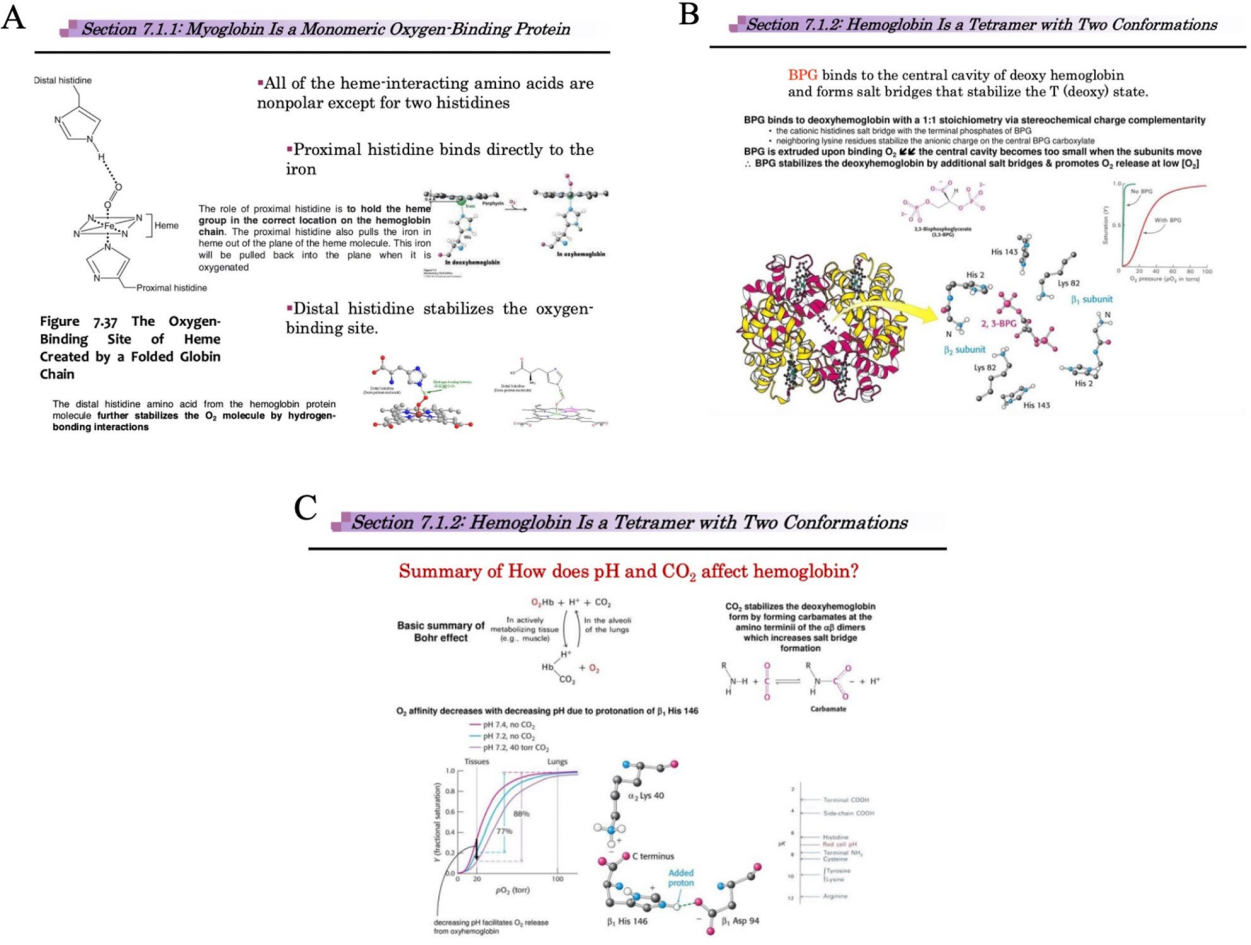
Lecture slides illustrating structural and regulatory mechanisms underlying hemoglobin’s conformational transition and oxygen-binding behavior (Chaari 2024). **A** Shows the coordination of Fe²⁺ within the heme group and the role of proximal and distal histidines in stabilizing oxygen binding. **B** Highlights the binding of 2,3-bisphosphoglycerate (2,3-BPG) to the central cavity of deoxyhemoglobin, forming salt bridges that stabilize the T state. **C** Explains the Bohr effect and the formation of additional salt bridges (e.g., His146–Asp94) under conditions of low pH or elevated CO₂, further stabilizing the deoxygenated state. These regulatory mechanisms were discussed in class to help students contextualize the structural dynamics they observed in the VR environment

To enhance students’ interpretation of structural features observed in VR, several foundational molecular concepts were reinforced in class lectures via the use of static visual aids. These included the coordination of the Fe²⁺ ion within the heme group, which is pulled into the plane of the porphyrin ring upon oxygen binding - a conformational change that initiates the T to R transition in hemoglobin. The role of the proximal histidine in anchoring the iron atom was emphasized, along with the distal histidine, which stabilizes bound oxygen through hydrogen bonding (Fig. 2A).

Also taught were regulatory mechanisms that stabilize hemoglobin’s T state, thus influencing oxygen affinity. For instance, 2,3-bisphosphoglycerate (2,3-BPG) binds to a central cavity between β-subunits in deoxyhemoglobin, where it forms electrostatic salt bridges with positively charged residues (e.g., His143, Lys82) (Fig. 2B). This interaction locks hemoglobin in the low-affinity T state, promoting oxygen release in peripheral tissues. Further, the Bohr effect and carbon dioxide (CO₂) sensitivity were discussed as additional allosteric modulators (Fig. 2C). Under acidic conditions or elevated CO₂ levels, additional salt bridges form (most notably between His146 and Asp94) which further stabilize the T state and decrease oxygen affinity. These cooperative mechanisms, introduced during the pre-VR orientation lecture, provided critical context for understanding the structural changes students subsequently explored in the immersive VR environment, linking molecular architecture with biological function.

#### Applied simulation & discussion (10 min)

In the final segment, students were encouraged to consolidate and articulate their structural insights through guided discussion and peer exchange. This phase emphasized reinforcing the spatial and biochemical concepts explored in VR such as subunit arrangement, heme group orientation, and the conformational changes associated with the T and R states of hemoglobin.

To support this, students participated in a brief interactive exercise, alternating roles as “presenters” and “questioners.” One student described a structural feature they had examined in VR (e.g., the heme pocket, subunit interfaces, or the α₁–β₂ contact point), while their partner asked clarifying or probing questions to challenge their understanding. The instructor similarly facilitated with reflective prompts such as “What visible changes did you notice between the T and R states?” or “How did the spatial orientation of subunits differ between the two conformations?” This dialogic format prompted students to explain structure–function relationships in their own words and helped reinforce key visual observations through peer discussion.

### Survey and data collection

To assess the educational impact of the intervention, a pre- and post-activity survey was administered. Anonymous surveys were completed in person immediately before and after the VR session using printed forms to ensure accessibility. This design allowed students to candidly reflect on their experience and enabled instructors to evaluate shifts in perception and understanding attributable to the VR-based activity. The instrument, developed specifically for this study (Figure S1), included both quantitative and qualitative components. Three core items were presented before and after the session, asking students to rate (on a 1–10 Likert scale) the perceived usefulness of VR in learning, their spatial understanding of the hemoglobin molecule, and their overall understanding of hemoglobin’s structure.

Post-activity, additional “yes/no” and open-ended questions captured user experience, engagement, comfort, and perceived pedagogical value of VR. Students were also asked to describe the most and least helpful aspects of the session, suggest other fields where VR could be applied, and reflect on potential biomedical research applications.

Survey completion rates were high: all 54 students completed the structured Likert-scale items, and 91% provided responses to open-ended questions. Data were anonymized prior to analysis.

### Data analysis

Quantitative data from the three pre- and post-activity Likert-scale items were analyzed descriptively. Responses were summarized using frequencies, percentages, and distributional shifts across predefined score ranges to characterize changes in student self-reported perceptions before and after the VR activity. Because survey responses were collected anonymously and paired identifiers were not retained, inferential paired statistical testing was not performed.

Binary “yes/no” responses were summarized as frequencies and percentages. Qualitative open-ended responses were analyzed thematically by two independent reviewers. Recurring patterns were grouped into categories such as “enhanced visualization,” “clarity of subunit structure,” “peer collaboration,” and “hardware challenges.” Representative student quotations were selected to illustrate each theme and to contextualize the quantitative findings.

## Results

### Participant characteristics and response rates

A total of 54 undergraduate medical students participated in the VR-based instructional session. All participants completed the structured pre- and post-activity Likert-scale survey items (100% response rate for quantitative measures). Responses to the open-ended qualitative questions (Q11–Q14) were provided by 49 students (91%).

Demographic characteristics such as age and gender were not collected, as the study was designed as an anonymous, classroom-based educational evaluation. Prior experience with virtual reality was assessed descriptively later in the survey (Q4-Q5). Information regarding visual acuity or the use of corrective lenses was not recorded as a study variable; students who routinely used glasses or contact lenses were permitted to wear them during the VR session, as described in the Methods section.

### Participant experience and reported discomfort

Student responses to Q10 indicated that most participants did not experience physical discomfort during the VR session (*n* = 35). Thirteen students reported transient discomfort at the beginning of the session, and six students reported persistent discomfort. No participants required medical attention, discontinued the VR activity due to discomfort, or withdrew from the session. All reported symptoms resolved spontaneously during or shortly after the activity.

The 9% non-response rate for open-ended survey items was not associated with withdrawal from the session or reported discomfort. All students completed the instructional activity in full, and no sessions were terminated early.

### Qualitative assessment of Clairty, Engagement, and peer learning

To examine changes in student perceptions following the VR activity, self-reported scores were compared descriptively across three domains: perceived usefulness of VR, spatial understanding of hemoglobin, and structural comprehension of the protein:

- Q1) *What is your perceived usefulness of Virtual Reality in learning coursework?* (1 = useless, 10 = essential)
- Q2) *How would you describe your spatial understanding of the hemoglobin molecule?* (1 = almost non-existent, 10 = mastery)
- Q3) *How would you describe your understanding of hemoglobin’s structure?* (1 = almost non-existent, 10 = mastery).

**Fig. 3:**
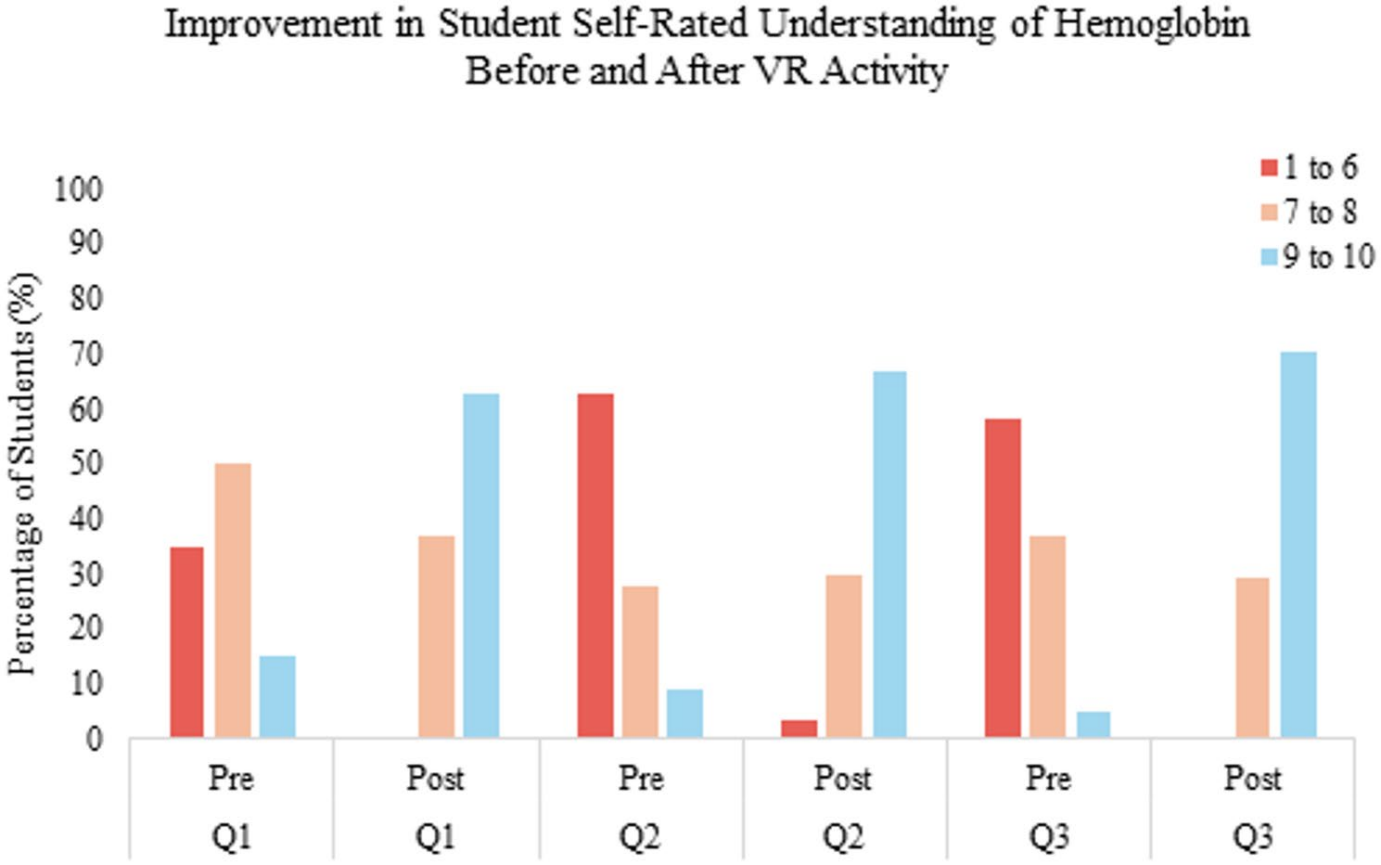
*Improvement in Student Self-Rated Understanding of Hemoglobin Before and After VR Activity (Q1-Q3).* Distribution of student self-rated scores across three core learning objectives before and after the virtual reality (VR) session using Nanome. Responses were grouped into score ranges of 1–6 (low understanding), 7–8 (moderate understanding), and 9–10 (high understanding or mastery). Each cluster compares pre- and post-session responses for questions on VR usefulness, spatial understanding of hemoglobin, and structural understanding of hemoglobin (*N* = 54). The proportion of students rating their understanding in the highest performance category (9–10) increased by 48.0% for perceived usefulness of VR (Q1), 57.7% for spatial comprehension of hemoglobin (Q2), and 65.5% for structural understanding of the protein (Q3)

Following the VR intervention, an upward shift was observed in self-reported scores across all three domains. The proportion of students rating their understanding in the highest performance category (9–10) increased by 48.0% for perceived usefulness of VR (Q1), 57.7% for spatial comprehension of hemoglobin (Q2), and 65.5% for structural understanding of the protein (Q3) (Fig. 3, Table S1). Results are presented descriptively to reflect changes in response distributions rather than inferential statistical comparisons.

### High rates of student endorsement and instructional value

To begin the post-activity survey, students were asked two general questions to contextualize their exposure to VR.

- Q4)Is this your first time using Virtual Reality (VR)?
  - No: 42     Yes: 12.
- Q5) If yes, how do you qualify the experience?
  - The same as before: 7    Better and new experience: 35.

**Fig. 4:**
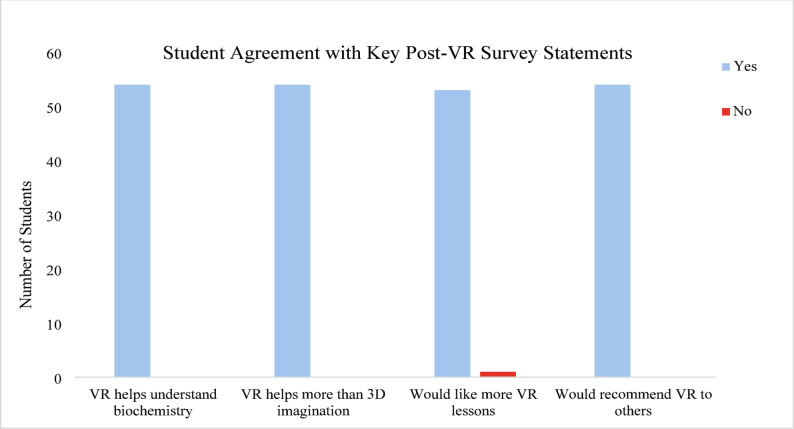
*Student Agreement with Key Post-VR Survey Statements (Q6-Q9)*. Student responses to selected post-session survey questions following the virtual reality (VR) activity. The majority of students agreed that VR enhanced their understanding of biochemistry concepts, was more effective than imagining molecular structures, and should be included in future sessions. Data represent the number of students responding “Yes” or “No” to each question (*N* = 54). All 54 students agreed that the VR session supported their understanding of biochemistry concepts, and 98% expressed interest in participating in future VR-integrated activities. 100% of respondents indicated that VR facilitated understanding beyond traditional 2D visual aids or internal mental modeling

Importantly, among the 42 students who had previously used VR, 35 still described this session as a “better and new experience” compared with prior encounters. Post-activity binary survey responses (Fig. 4, Table S2) indicated high levels of student endorsement. All 54 students agreed that the VR session supported their understanding of biochemistry concepts, and 98% expressed interest in participating in future VR-integrated activities. All respondents indicated that VR facilitated understanding beyond traditional 2D visual aids or internal mental modeling.

### Clarity, Engagement, and peer learning

Student feedback from open-ended questions highlighted several recurring themes, most frequently emphasizing how features such as molecular rotation, subunit differentiation, and detailed exploration of heme interactions improved their spatial understanding and engagement with the content (Table 1). This visual clarity was followed closely by themes of interactivity and peer-based engagement. As one student noted, “I was able to observe each subunit very closely, and it was much clearer than photos I can find on the internet.”

**Table 1:** *Thematic summary of student reflections on the VR session based on Open-Ended feedback (Q11–Q14).* Themes were derived from qualitative analysis of open-ended responses. Frequencies are approximate and based on recurring patterns across multiple student submissions (*N* = 30). Example quotes are representative of each theme

Theme	Example Quote	Frequency
Enhanced molecular visualization	“Being able to rotate the hemoglobin molecules to see different angles”	6+
Increased engagement & interactivity	“How hands-on it was”; “Seeing a 3D version of what we talked about in class”	5+
Clarity of subunit structure	“I was able to observe each subunit very closely; much clearer than photos”	3+
Social/collaborative learning	“Taking role in explaining 3D structure to my colleague”	2+
Minor discomforts	“The headset was a bit blurry”; “It was short. I hope to do it again.”	6+

Another student highlighted the collaborative benefit of the session: “Taking a role in explaining 3D structure to my colleague helped me understand it better myself.”

While some students reported discomfort due to headset fit or session length, these issues were peripheral and hardware-related rather than content-driven. Importantly, they did not detract from the perceived educational value of the experience. In fact, some of the most common descriptors students used to characterize their experience with VR. “Fun,” “helpful,” and “useful” were the most frequently cited, suggesting that the session simultaneously satisfied both affective and cognitive learning dimensions (Table S3). These terms were commonly used across responses to characterize the learning experience.

Furthermore, the positive lexical framing used by students in Q14 responses reinforces the notion that VR can bridge the gap between engagement and rigor - a balance that traditional didactic modalities often struggle to achieve. This was complemented by responses to two critical thinking questions, which asked students to apply structural insights from the VR session to novel contexts.

In Q15, students were asked:

"How could future drugs be designed to target the polymerization of hemoglobin in sickle cell anemia? Based on what you observed in Nanome and your knowledge, what interactions would they aim to disrupt?"

This question built on earlier class discussions of hemoglobin pathology, linking lecture-based concepts with the VR-based exploration of subunit interfaces. By visualizing the non-covalent interactions stabilizing hemoglobin’s quaternary structure, such as those between α₁-β₂ and α₂-β₁ contacts, students were better equipped to identify potential molecular targets for therapeutic intervention. Their responses demonstrated clear application of spatial and mechanistic reasoning, with suggestions to disrupt hydrophobic aggregation surfaces or stabilize the R state to prevent polymer formation. This integration of structural visualization with biochemical knowledge supported more meaningful critical thinking around disease mechanisms and drug design.

In Q16, students were asked:

“In which fields other than Biochemistry could you see a similar approach being useful for teaching difficult or abstract concepts?”

Most students identified spatially intensive subjects such as anatomy, organic chemistry, general chemistry, and physiology as areas in which a similar VR-based approach could support learning of abstract concepts. Responses emphasized the value of three-dimensional visualization for understanding stereochemistry, molecular orientation, and anatomical relationships (Table S3).

## Discussion

### Immersive visualization and structural Understanding in biochemistry

This study contributes to the evolving discourse on immersive learning technologies by demonstrating that VR can facilitate concept mastery and structural reasoning in molecular biology. Traditional instruction in biochemistry relies heavily on 2D representations to teach inherently 3D molecular phenomena. However, these static resources are often insufficient for conveying dynamic concepts such as allosteric transitions or quaternary interactions [8, 9]. Empirical studies have shown that learners frequently struggle to mentally reconstruct molecular forms from such 2D tools, resulting in conceptual gaps and misinterpretations [10, 11]. This challenge is particularly acute in the study of macromolecular systems like hemoglobin, where spatial orientation and conformational state transitions are central to biological function [12].

The Nanome VR platform provides a solution by offering real-time, manipulable molecular environments derived from structural databases such as the PDB. Through immersive visualization of PDB structures like 1A3N and 6BB5, learners can rotate, scale, and explore protein subunits, observe ligand binding pockets, and track domain-specific movements during state transitions. The design of Nanome supports multisensory engagement and direct manipulation of atomic variables, which allows for a comprehensive interaction model where students actively probe spatial features instead of passively interpreting 2D projections [13]. The structure of such models aligns with the concept of cognitive load theory, which explains the usage of certain tools allows for offloading of mental reconstruction tasks thereby enabling learners to focus on interpretive reasoning [14, 15].

Within this study, students reported improved clarity in understanding hemoglobin’s conformational shifts and cooperative binding properties. While these findings reflect self-reported perceptions rather than objective performance measures, they are consistent with prior research describing the spatial learning advantages of immersive environments in molecular and biomedical education.

### Engagement, collaboration, and conceptual integration

The Nanome platform’s multi-user design further enhanced conceptual engagement by supporting synchronous collaboration. Students were able to co-inhabit molecular environments with peers and instructors, mark regions of interest, and co-navigate the structural landscape. This feature encourages collaborative exploration, where students can explain ideas, ask questions, and correct misunderstandings, all processes that drive deeper understanding [16, 17]. Such collaborative environments replicate the dynamics of scientific teamwork, mirroring how molecular biologists and clinicians interrogate data in real-world settings.

In addition to cognitive outcomes, students reported increased confidence in reasoning through complex structures (Table 1). These affective dimensions of learning are critical, as confidence and perceived competence are predictors of persistence in STEM fields [18, 19]. Prior work has also shown that immersive VR can enhance motivation by fostering a sense of presence and agency, especially when learners have control over their exploratory path [16, 20]. In our study, students not only retained structural facts but used them to explain and infer function, indicating that VR supported the transition from surface-level familiarity to integrated understanding.

Conceptual integration is further strengthened by VR’s ability to align structure with function in a coherent spatial narrative. Hemoglobin, as a model protein, presents an ideal case for this, as oxygen-binding dynamics, subunit cooperativity, and pathological mutations all manifest structurally [21, 22]. Through immersive interaction, students were able to track shifts between R and T states, visualize how a single mutation could alter the geometry of a binding site, and predict downstream effects.

In contrast to other educational technologies, VR does not abstract structure away from interaction. It places students inside the molecular context, allowing learning to unfold through engagement with authentic, high-resolution data [23, 24]. Nanome’s ability to render complex protein assemblies with real-time responsiveness provides a level of granularity that enhances accuracy in molecular reasoning. This feature is crucial in upper-educational and graduate-level courses, where learning objectives extend beyond recognition and into hypothesis generation, mechanistic modeling, and translational thinking. However, given the short duration of the intervention and the absence of delayed assessment, these findings should be interpreted as perceived conceptual integration rather than demonstrated long-term retention.

### Instructor reflections & limitations

While student responses provided valuable insight into the perceived learning gains and effectiveness of the VR activity, it is equally important to consider the experience from the instructor’s perspective. Implementing immersive VR in a live educational setting offered clear opportunities to enhance structural understanding and engagement but also introduced logistical and instructional challenges that shaped the design and delivery of the session.

One of the most significant benefits observed was the ability to dynamically illustrate molecular complexity - particularly hemoglobin’s T to R conformational shift - through real-time 3D manipulation. Students could visually inspect key structural features, such as subunit interfaces, heme orientation, and salt bridge interactions, from multiple angles. This hands-on spatial interaction facilitated student interpretation of abstract concepts that often remain inaccessible through traditional 2D diagrams, echoing the spatial learning gains reported in other molecular modeling studies using immersive media [25, 26].

Instructors also noted an increase in student participation, especially during the peer role-play portion of the session. This structure prompted students to explain molecular features in their own words and respond to peer questions in real time, resulting in stronger engagement and collaborative sense-making. Prior work supports this observation: VR environments have been shown to encourage active verbalization, deeper conceptual discussion, and improved motivation when used in small-group or dialogic formats [27–29].

However, several limitations were identified. First, the implementation required access to reliable VR hardware (Oculus Quest 2 headsets), a functioning local network, and sufficient technical support. While most sessions ran smoothly, there were instances of connection delays and interface troubleshooting, consistent with challenges reported in other STEM VR applications [30, 31]. Additionally, onboarding students to Nanome took time, and a few participants experienced mild discomfort, such as eye strain or disorientation - effects that are well documented in immersive technology literature [32].

Instructor preparation was also a significant factor. Selecting suitable molecular files, preloading environments, developing targeted discussion prompts, and aligning the session with prior lecture content required more effort than typical lecture-based formats. Research suggests that VR is most impactful when tightly integrated into the curriculum and supported by clear learning objectives [28, 33]. In this session, having students encounter key concepts - such as hemoglobin subunit interactions and allosteric regulation - during lecture before the VR component proved essential for depth of engagement.

Finally, the study was limited in scope. It focused on a single protein target, was delivered in a one-time session, and involved a relatively small student cohort. While feedback was overwhelmingly positive, further investigation is needed to evaluate the long-term retention of structural concepts, the efficacy of repeated VR exposure, and the feasibility of scaling this approach across other biochemical topics or larger courses [27, 31].

## Future applications

As VR becomes more established in science education, the next step is to develop systems that deliver lasting educational value and practical relevance. Effective VR applications should support adaptive learning, generate actionable insights through analytics, and reflect the complexity of real-world biomedical research and industry practices. The following section outlines key areas for growth, including intelligent tutoring systems, cross-disciplinary collaboration, scalable classroom integration, and high-fidelity industrial simulations.

One particularly promising avenue is the integration of artificially intelligent (AI) tutoring systems within VR environments. These adaptive agents can respond to user behavior, provide scaffolded support, and highlight relevant structural features as students navigate the molecular space. Such AI-guided instruction has been proposed as a necessary evolution to address cognitive variability and reduce user frustration in autonomous VR learning settings [34]. When paired with learning analytics, these environments could also generate actionable data for formative assessment and instructional feedback [35].

In addition to informing real-time feedback, the data generated through learning analytics can also support more rigorous evaluations of long-term effectiveness, highlighting the need for future studies to go beyond immediate post-session metrics and incorporate delayed assessments, behavioral retention, and transfer-based measures. Evidence suggests that immersive VR leads to superior long-term retention compared to traditional formats [16], and enhances performance in applied tasks such as safety inspections and procedural simulations [36]. VR-based skill acquisition has also been shown to generalize to real-world settings, indicating strong potential for transfer of learning [37]. However, this gap in longitudinal research limits our understanding of whether immersive learning translates into durable cognitive outcomes or improved long-term performance in biochemistry and related disciplines. Randomized controlled trials comparing VR-enhanced instruction with non-VR approaches would be particularly valuable in establishing the educational impact of immersive molecular visualization in medical biochemistry.

Existing literature increasingly emphasizes the importance of developing VR educational modules that closely align with real-world biomedical goals, including disease modeling, molecular interaction networks, and cellular pathogenesis. GeneNet VR exemplifies this shift by offering an immersive, interactive platform for exploring large-scale gene-to-gene interaction networks, such as those derived from breast cancer datasets [38]. Unlike traditional 2D visualization systems that suffer from visual congestion and network entanglement [39] - where densely connected nodes and edges lead to interpretability loss - GeneNet VR leverages the spatial depth and intuitive navigation of standalone VR hardware like the Oculus Quest to present high-dimensional data in a more coherent and accessible format. Biomedical researchers found the platform not only accessible for a wide range of users but also valuable for identifying clusters and co-expression relationships relevant to disease processes [38]. This suggests that scalable, affordable VR tools can play a transformative role in education and analysis, particularly when they support performance benchmarks and are aligned with real research applications in molecular systems biology.

From an institutional perspective, scalable implementation will require both platform interoperability and instructor readiness. Faculty development programs should not only focus on navigating VR technology but also emphasize instructional design strategies that use immersive tools to meet specific learning outcomes [34]. Furthermore, embedding VR into active learning environments, such as flipped classrooms, problem-based learning (PBL) modules, and capstone design projects, can enhance hybrid instructional strategies by promoting deeper engagement, student autonomy, and real-world skill development simultaneously [29, 31, 40]. These interactive formats are well-suited to VR’s strengths in spatial visualization and experiential learning, making complex molecular processes more tangible. Furthermore, cross-disciplinary VR experiences where students from biochemistry, bioinformatics, and pharmacology collaborate in shared virtual environments can foster a more integrated, systems-level understanding of molecular biology and disease mechanisms [35]. Such approaches not only reinforce conceptual links between disciplines but also simulate the collaborative nature of biomedical research and clinical problem-solving.

Considerations of equity and access are critical when evaluating immersive educational technologies such as virtual reality. While standalone headsets and commercial VR platforms are becoming more widely available, implementation remains constrained by institutional resources, infrastructure, and technical support requirements [3]. In addition, VR-based learning activities are often delivered in facilitated, small-group settings that require instructor presence, scheduling flexibility, and dedicated hardware, conditions that may not be uniformly accessible across educational contexts. Prior reviews have cautioned that unequal access to immersive technologies may exacerbate existing disparities if adoption occurs without parallel attention to scalability, cost-effectiveness, and inclusive instructional design [4, 7]. Future research should therefore evaluate not only pedagogical outcomes, but also equity, sustainability, and access when assessing the educational impact of VR in biomedical curricula.

Industry-aligned applications of VR in education merit deeper exploration, particularly as workforce demands in biotechnology and pharmaceutical sectors grow more complex and interdisciplinary. Immersive VR environments can be leveraged to simulate real-world workflows, such as biochemical manufacturing, protein purification and engineering pipelines, or drug target validation procedures, offering students exposure to industrial processes typically inaccessible in conventional classrooms [41]. These high-fidelity simulations can reinforce the practical application of core scientific principles while enhancing decision-making, troubleshooting, and procedural fluency. For example, VR-based molecular modeling tools have been shown to improve spatial reasoning and understanding of protein-ligand interactions, key skills in drug design and development [38, 42]. By enabling students to engage directly with complex systems in risk-free, interactive settings, VR acts as a bridge between conceptual mastery and industry-specific competencies. In this way, VR does not merely support theoretical understanding, but serves as a tool for professional preparation, aligning academic training with the operational standards and collaborative dynamics found in biopharma, bioinformatics, and biomedical engineering fields [43].

## Conclusion

This study explored the use of an immersive VR platform (Nanome) to support undergraduate medical students’ understanding of hemoglobin structure and function in a preclinical biochemistry course. Through a structured VR session, students interacted with three-dimensional molecular models and completed pre- and post-activity surveys assessing their perceptions of understanding and engagement.

Students reported improved clarity in visualizing molecular structure, high levels of engagement, and positive perceptions of VR as a supplementary instructional tool. While these findings are based on self-reported data and reflect short-term perceptions, they suggest that immersive VR may offer a valuable complement to traditional teaching methods for conceptually dense and spatially complex topics in biochemistry. Further research using controlled designs and objective learning outcomes is needed to determine the impact of VR-based instruction on long-term learning and academic performance.

## Supplementary Information

Supplementary Material 1.

Supplementary Material 2.

## Data Availability

The datasets generated and analyzed during the current study are not publicly available due to institutional restrictions and participant privacy considerations. De-identified data may be made available from the corresponding author on reasonable request and subject to IRB approval.

## Abbreviations

2D: Two-dimensional
3D: Three-dimensional
AI: Artificial Intelligence
CAMIL: Cognitive Affective Model of Immersive Learning
CO2: Carbon Dioxide
Fe²⁺: Ferrous ion
Hb: Hemoglobin
HMD: Head-Mounted Display
IRB: Institutional Review Board
O₂: Oxygen
PBL: Problem-Based Learning
PDB: Protein Data Bank
R state: Relaxed (oxygenated) state of hemoglobin
T state: Tense (deoxygenated) state of hemoglobin
VR: Virtual Reality
WCM-Q: Weill Cornell Medicine–Qatar
