# Supplementary material for: Immersive virtual reality for teaching hemoglobin structure in preclinical medical biochemistry education: a mixed-methods study of student self-reported perceptions
Source: BMC Med Educ. 2026 Feb 3;26:372. doi: 10.1186/s12909-026-08736-4 (PMC12958723; doi:10.1186/s12909-026-08736-4)
Supplement: Supplementary file 1 — Supplementary Material 1. [file 12909_2026_8736_MOESM1_ESM.pdf]

Exploring Hemoglobin Structure and Function Using Nanome

Assessment Questions:

A. Pre-Activity Reflection

1- What is your perceived usefullness of Virtual Reality in learning coursework? (1-10 ; 1 = useless, 10 = essential)

2- How would you describe your spatial understanding of the hemoglobin molecule? (1-10 ; 1 = almost non-existent, 10 = mastery)

3- How would you describe your understanding of hemoglobin's structure? (1-10 ; 1 = almost non-existent, 10 = mastery)

B. Post-Activity Reflection

1- What is your perceived usefullness of Virtual Reality in learning coursework? (1-10 ; 1 = useless, 10 = essential)

2- How would you describe your spatial understanding of the hemoglobin molecule? (1-10 ; 1 = almost non-existent, 10 = mastery)

3- How would you describe your understanding of hemoglobin's structure? (1-10 ; 1 = almost non-existent, 10 = mastery)

4- How interacting with hemoglobin in VR enhanced your understanding of protein structure?

Generated on IRBNet

C- VR related questions:

1- Is it the first time that you use The Virtual Reality (VR)

No
Yes

2- If yes, how do you qualify the experience?

- The same as before
- Better and new experience

3- Thinking back to using the VR technology, tell me your initial reaction to using it?

4- What did you like the most about the session?

5- What did you like least about the session?

6- VR can helps to better understand some of the Biochemistry concepts taught in the class.

No
Yes

7- Seeing proteins in three dimensions can help me to understand something that I didn't understand with a two-dimensional representation.

No
Yes

8- Do you think it will help to better understand the difference differences between the protein structures, interaction between protein and substrates, between A-DNA, B-DNA and Z-DNA….

Generated on IRBNet

No
Yes

9- VR helps to learn better the molecule structures and interactions than using imagination of 3D structures in my brain.

No
Yes

10- How is you experience with The Virtual Reality (VR) protein part?

11- The Nanome application has great potential for teaching chemistry concepts.

No
Yes

12- I experienced physical discomfort while participating in the VR lesson.

- No
- Only at the begging
- Yes

13- What aspects of the VR experience did you find problematic? (Multiple choices)

14- I would like to participate in more VR lessons.

No
Yes

15- I would recommend VR lessons to other students.

Generated on IRBNet

No
Yes

16- Please list three words or phrases you would use to describe VR technology in education

17- Additional feedback

C. Critical Thinking Questions:

Q1: "How could future drugs be designed to target the polymerization of hemoglobin in sickle cell anemia? Based on what you observed in Nanome and your knowledge, what interactions would they aim to disrupt?"

Q2: "How do you think VR technology could be used in other areas of biomedical education or research to enhance the learning experience?"

Generated on IRBNet
