# Supplementary material for: Immersive virtual reality for teaching hemoglobin structure in preclinical medical biochemistry education: a mixed-methods study of student self-reported perceptions
Source: BMC Med Educ. 2026 Feb 3;26:372. doi: 10.1186/s12909-026-08736-4 (PMC12958723; doi:10.1186/s12909-026-08736-4)
Supplement: Supplementary file 2 — Supplementary Material 2. [file 12909_2026_8736_MOESM2_ESM.docx]

**Table S1.** *Percentage Distribution of Student Self-Ratings Across Three Key Questions Before and After the VR Session (Q1-Q3).* Responses were categorized into three score ranges (1–6 = low, 7–8 = moderate, 9–10 = high). Values represent the percentage of students selecting each score range for each question (N = 54).

| Question | Timepoint | 1-6 (%) | 7-8 (%) | 9-10 (%) |
| --- | --- | --- | --- | --- |
| Q1 | Pre | 35.0 | 50.0 | 15.0 |
| Q1 | Post | 0.0 | 37.0 | 63.0 |
| Q2 | Pre | 63.0 | 28.0 | 9.0 |
| Q2 | Post | 3.7 | 29.6 | 66.7 |
| Q3 | Pre | 58.0 | 37.0 | 5.0 |
| Q3 | Post | 0.0 | 29.5 | 70.5 |

**Table S2**. *Student Responses to Key Post-VR Survey Questions (Q6-Q9).* Values represent the number of students selecting each response. Questions reflect perceived educational value and willingness to engage with VR in future coursework (N = 54).

| Prompt | Yes | No |
| --- | --- | --- |
| VR can help me to better understand some of the Biochemistry concepts taught in the class. | 54 | 0 |
| VR can help me learn the molecular structures and interactions better compared to the imagination of 3D structures in my brain. | 54 | 0 |
| I would like to participate in more VR lessons. | 53 | 1 |
| I would recommend VR lessons to other students. | 54 | 0 |

**Table S3**. Frequencies of Words Used by Students to Describe the Virtual Reality Session in Biochemistry Education (Q11-Q14). Words were collected from 30 student responses to an open-ended prompt asking for three words or phrases describing VR in an educational context. Frequencies reflect total mentions across all responses.

| **Word** | **Frequency** |
| --- | --- |
| Interactive | 6 |
| Informative | 6 |
| Interesting | 6 |
| Useful | 9 |
| Helpful | 9 |
| Fun | 18 |
